# *Bifidobacterium animalis* subsp. *lactis* Probio-M8 undergoes host adaptive evolution by *glc*U mutation and translocates to the infant’s gut via oral-/entero-mammary routes through lactation

**DOI:** 10.1186/s40168-022-01398-6

**Published:** 2022-11-22

**Authors:** Zhi Zhong, Hai Tang, Tingting Shen, Xinwei Ma, Feiyan Zhao, Lai-Yu Kwok, Zhihong Sun, Menghe Bilige, Heping Zhang

**Affiliations:** 1 Key Laboratory of Dairy Biotechnology and Engineering, Ministry of Education, Inner Mongolia Agricultural University, Hohhot, 010018 China; 2 Key Laboratory of Dairy Products Processing, Ministry of Agriculture and Rural Affairs, Inner Mongolia Agricultural University, Hohhot, 010018 China; 3 Inner Mongolia Key Laboratory of Dairy Biotechnology and Engineering, Hohhot, 010018 China

**Keywords:** Bifidobacteria, Gut microbiota, Mother-to-infant bacterial transmission, Lactation, Adaptive evolution, *glc*U

## Abstract

**Background:**

Most previous studies attempting to prove the phenomenon of mother-to-infant microbiota transmission were observational, performed only at genus/species-level resolution, and relied entirely on non-culture-based methodologies, impeding interpretation.

**Results:**

This work aimed to use a biomarker strain, *Bifidobacterium animalis* subsp. *lactis* Probio-M8 (M8), to directly evaluate the vertical transmission of maternally ingested bacteria by integrated culture-dependent/-independent methods. Our culture and metagenomics results showed that small amounts of maternally ingested bacteria could translocate to the infant gut via oral-/entero-mammary routes through lactation. Interestingly, many mother-infant-pair-recovered M8 homologous isolates exhibited high-frequency nonsynonymous mutations in a sugar transporter gene (*glc*U) and altered carbohydrate utilization preference/capacity compared with non-mutant isolates, suggesting that M8 underwent adaptive evolution for better survival in simple sugar-deprived lower gut environments.

**Conclusions:**

This study presented direct and strain-level evidence of mother-to-infant bacterial transmission through lactation and provided insights into the impact of milk microbiota on infant gut colonization.

Video Abstract

**Supplementary Information:**

The online version contains supplementary material available at 10.1186/s40168-022-01398-6.

## Background

The infant gut microbiota plays an important role in the development of the immune, metabolic, and nervous systems [1–3]. However, the formation of infant gut microbiota and factors causing individual differences are still not completely understood. In view of the natural bond between mother and infant, maternal microbiota, including those from the mother’s gut, skin, vagina, and saliva [4–6], are considered to be the major source of infant microbiota [7], even though there are still many controversies about when and in what way the mother’s microbiota is transferred to the infant [8]. Recent reports have revealed that bacteria are present in the mother’s placenta, umbilical cord, and amniotic fluid, strongly supporting that a pre-birth infant gut microbiome might have existed even before delivery [9–11], though the hypothesis is still under debate [12, 13]. In addition, many perinatal conditions, e.g., mode of delivery, type of feeding, and antibiotic usage, could also affect infants’ gut microbiota [4, 14]. However, it is generally believed that infants’ gut microbiota are drastically reshaped in the first years of life until the age of three when the gut microbiota become mature [15]. Thus, it would be of interest and indeed crucial to grasp the window of opportunity for programming the gut microbiota, especially through maternal diet and lactation.

It has been reported that gut microbiota can be vertically transmitted from mother to infant through lactation, but most studies are observational and have been done on a relatively low taxonomic resolution limiting to the genus and/or species levels [16–19]. Moreover, due to the large amount of overlapping between microbiota normally present in the gut, vagina, and breast milk, research outputs produced on the genus and species levels lack specificity [7]. These issues hinder accurate interpretation and further development of strategies for target modulation of infant gut microbiota. Even though recent advances in metagenomic technologies have enabled the design of studies for investigating mother-to-infant bacterial transmission by deep sequencing, providing further observational evidence for such phenomenon [6, 20], it would still be necessary to fully validate metagenomic findings via integrative functional analyses like laboratory cultivation, isolation, and biochemical characterization of specific strains [7].

The objective of this work was to provide direct evidence for the vertical transmission of bacteria from mother to infant via maternal bacteria intake during lactation. A biomarker strain, *Bifidobacterium* (*B*.) *animalis* subsp. *lactis* Probio-M8 (M8), was selected for tracking the mother-to-infant transmission through breast milk. A combination of methods, including laboratory cultivation and identification, strain-level metagenomics, and Phenotype MicroAssay analysis, was employed to detect and recover M8 homologous isolates from the M8 dry powder given to the mothers, as well as the breast milk and fecal samples of mother-infant pairs. The diversity, genetic variations, and carbon utilization preference and capacity of the isolates were further analyzed. The long-term goal of this study was to provide guidance for probiotic intervention in the programming of infant gut microbiota, so as to improve human health.

## Methods

### Bacterial strain

The M8 strain was obtained from the Lactic Acid Bacteria Collection Centre of Inner Mongolia Agricultural University of China. It was a human breast milk-originated strain [21]. Bacteria were propagated anaerobically (80% N_2_, 10% H_2_, and 10% CO_2_) in Reinforced Clostridial Medium (RCM; HopeBio, Qingdao, China) at 37°C.

### Study design and sample collection

A total of 11 healthy mother-infant pairs during lactation were recruited for this study (Supplementary Table 1). Exclusion criteria were preterm, any formula feeding, drug administration during the neonatal period (mother and/or neonate), and any variables known to affect the balance of the maternal and/or neonatal microbiota, such as gastrointestinal and immunological disorders. During the trial, the lactating mothers ingested M8 dry powder daily in the form of individually packaged sachets (one sachet per day, containing 6×10^10^ cfu M8). The M8 dry powder was produced in-house in a small batch production complying with the Good Manufacturing Practice guidelines. Unfortunately, this study could not include a control group of subjects without M8 ingestion due to its overlapping time frame with the start of the COVID-19 pandemic, making it even harder to recruit eligible mother-infant pairs. All procedures involving human subjects were approved by the Ethics Committee of the Affiliated Hospital of Inner Mongolia Medical University (Project number: KY(2020011)). Written informed consent was obtained from all adult participants.

Breast milk, feces of mothers and infants were collected continuously once or twice a week for 8–15 weeks after starting the M8 intervention (Supplementary Table 1). Fresh feces (10–15 g) were collected into 50-mL sterile sampling tubes. The breast and nipple area were cleaned with aseptic soap before expressing milk. Breast milk was collected using a sterile electrical breast pump after discarding the foremilk. Ten milliliters of breast milk was collected into 15-mL sterile sampling tubes. Samples were transported at 4°C and processed within 2 h in an anerobic chamber with an atmosphere of 80% N_2_, 10% H_2_, and 10% CO_2_. Aliquots of breast milk (0.5 mL) and feces (0.5 g) samples were immediately subjected to culture, and the remaining portions of samples were mixed with a sample protector for RNA/DNA (TaKaRa, Shiga, Japan) and stored at −80°C prior to DNA extraction and metagenomic sequencing.

### Culture and isolation of *B. animalis* subsp. *lactis* from breast milk and fecal samples

Breast milk samples (each of 0.5 mL) were diluted to 10^-1^ and 10^-2^ in phosphate buffer saline (PBS; 8.0 g of NaCl/L, 0.2 g of KH_2_PO_4_/L, and 1.15 g of Na_2_HPO_4_/L; pH 7.2). Fecal samples (each of about 0.5 g) were diluted to 10^-5^ and 10^-6^ in PBS. Then, 200 μL of each diluted breast milk and fecal sample was spread on triplicate sets of RCM agar plates and incubated anaerobically (80% N_2_, 10% H_2_, and 10% CO_2_) at 37°C for 72 h to target *B*. *animalis* subsp. *lactis*. Colonies with morphological characteristics of *B*. *animalis* subsp. *lactis* were isolated by streaking for purity and cultured in liquid media. The purity of isolates was confirmed microscopically before centrifugation (8000 g, 10 min) to collect cell pellets to be stored at −20°C prior to DNA extraction. Meanwhile, viable cells were cryopreserved at −80°C in 20% (v/v) glycerol.

### Isolation of *B. animalis* subsp. *lactis* from M8 dry powder

Prior to the trial, two sachets of M8 dry powder were randomly chosen among the M8 products to be distributed to the participants for consumption. The bacteria powder in these sachets were resuspended and thoroughly vortexed in PBS. The mixture was then streaked on RCM agar plates and incubated anerobically (80% N_2_, 10% H_2_, and 10% CO_2_) at 37°C for 72 h. Twenty-four colonies were re-streaked to purity and cultured in liquid media. The purity of isolates was confirmed microscopically before centrifugation (8000 g, 10 min) to collect cell pellets to be stored at −20°C prior to DNA extraction. Meanwhile, viable cells were cryopreserved at −80°C in 20% (v/v) glycerol.

### DNA extraction for 16S rDNA sequencing and whole genome sequencing

Frozen cell pellets of each isolate were thawed, and the total DNA was extracted using the TIANamp Bacteria DNA Kit (Tiangen, Beijing, China) according to the manufacturer’s instructions. The concentration and purity of extracted DNA were checked by a Nanodrop spectrophotometer (Thermo Fisher, Madison, USA).

### Identification of isolates by 16S rDNA sequencing

Purified DNA (50 μL) was diluted to the concentration of 100 ng/μL for 16S rDNA amplification. The primers (forward (5′-GGGTGGTAATACCGGATG-3′) and reverse (5′- GACCATGCACCACCTGTGAA-3′)) targeting *B. animalis* subsp. *lactis* 16S rDNA were designed by Primer Premier 5.0. The 16S rDNA sequencing was performed by Majorbio Bio-Pharm Technology Corp. (Shanghai, China). A sequence homology alignment search was performed using BLAST of NCBI (https://blast.ncbi.nlm.nih.gov/Blast.cgi) to confirm the identity of each isolate.

### Whole-genome sequencing of individual isolates

Deep sequencing of the whole genome of all *B*. *animalis* subsp. *lactis* isolates was performed on the Illumina HiSeq platform (Illumina Inc., San Diego, CA, USA) by generating 150 bp paired-end libraries. At least 1.0 GB clean data was obtained for each genome. Paired-end reads were first assembled de novo using SOAPdenovo v1.06 [22]. GapCloser (http://sourceforge.net/projects/soapdenovo2/files/GapCloser/) was used to fill local inner gaps and correct single base errors. Glimmer v3.02 was used to predict putative coding sequences [23]. Kyoto Encyclopedia of Genes and Genomes (KEGG) [24] and Clusters of Orthologous Genes (COG) [25] databases were used for functional annotation of predicted open reading frames (ORFs).

### Construction of core-genome

All predicted ORFs were classified to their corresponding gene families according to methods described in a previous study [26], and all identified gene families were included for core-genome construction. The SiLiX software [27], which categorizes detected genes into defined homologous gene families, was used to build the core-genome.

### Construction of phylogenetic trees

Core-gene phylogenetic trees of *B*. *animalis* subsp. *lactis* were constructed. Core gene nucleotide sequences were aligned by using an online software, MUSCLE v3.8.31 [28]. Gblocks and Gubbins were applied to remove regions of ambiguous alignment and intragenic homologous recombination, respectively [29]. Then, FastTree 2.1.8 [30] was used to construct phylogenetic trees using the maximum likelihood method (with 1000 bootstrap iterations) from the concatenated alignments. The phylogenetic trees were visualized with Interactive Tree Of Life (iTOL) [31].

### Identification of genomic structural variations

Genomic structural variations of M8 homologous isolates were identified by comparing against the complete genome sequence of M8 (retrieved from the GenBank database under the accession number CP047190). Paired-end reads generated in this study were first mapped to the genome of M8 by BWA [32], then SAMtools was used to identify single nucleotide polymorphisms (SNPs) and insertions and deletions (InDels) [33]. The identified SNPs and InDels were filtered using the following criteria: (a) > 20-fold coverage of paired-end reads, (b) ratio of reads supporting an SNP or InDel > 0.7, and (c) not in repetitive regions.

### Verification of mutations

The SNPs identified in the *glc*U gene were verified by PCR with the primers: forward (5′-TCGACGGCAAGCCAAGTCAG-3′) and reverse (5′-ATCGCCATAAGCACCGCACC-3′). Amplified DNA fragments were sequenced by Majorbio Bio-Pharm Technology Corp., Shanghai, China.

### Metagenomic sequencing, assembly, binning, and annotation

Representative maternal and infant fecal samples (collected after four and eight weeks of M8 intake) were subjected to deep metagenomic sequencing. The DNA of maternal and infant feces (around 100 mg per sample) was extracted using the QIAamp Fast DNA Stool Mini Kit (Qiagen, Hilden, Germany). Metagenomic sequencing was processed using the Illumina Hiseq Xten platform (Illumina, Inc., San Diego, CA, USA). The obtained 150 bp paired-end raw reads were first quality-trimmed using Trim Galore (http://www.bioinformatics.babraham.ac.uk/projects/trim_galore/) with default settings. At least 50 GB clean data was obtained for each sample. Clean reads of individual samples were assembled de novo using MetaSPAdes [34] with the parameter “-meta -k 21,33,55,77”. Scaffolds were organized into genome bins based on tetranucleotide frequency and sequence coverage using Maxbin v2.2.3 [35]. Completeness and contamination levels of metagenome-assembled genomes (MAGs) were estimated based on the representation of lineage specific tRNAs and marker gene sets using CheckM v1.0.5 [36]. The taxonomy of reconstructed MAGs was determined using CAT v5.0.3 [37]. Genes in the reconstructed MAGs were predicted and translated to proteins using Prodigal v2.6.3 [38] before being annotated using BLAST against the NCBI-nr database.

### Phenotype MicroArray analysis

The preference and capacity of carbon source utilization of the isolates were analyzed using Phenotype MicroArray (PM) Technology (Biolog, Inc., Harvard, CA, USA) on AN MicroPlateTM designed for identification and analysis of anerobic bacteria. Cryopreserved isolates were reactivated by two rounds of streaking on RCM agar plates for single colonies (cultured for 72 h in each round), and freshly grown colonies were picked for bacterial phenotype identification following the manufacturer’s instructions for anaerobic bacteria (no dye). Bacteria were added to the inoculating fluid AN, and the transmittance was measured and adjusted to 65%. Cells suspended in inoculating fluid AN were dispensed with an automatic multichannel pipettor into AN 96-well MicroPlates (100 μL per well). The AN MicroPlates were packed in plastic bags with oxygen absorber, heat sealed immediately, incubated at 37 °C for 72 h, and read using the MicroLog plate reader and associated software. Changes in the absorbance during incubation were resulted from the reduction of tetrazolium dye by respiring cells, which were measured at 15 min intervals by the OmniLog system. The monoculture growth curve and the area under the curve (AUC), representing the growth and preference of carbon source utilization of individual isolates, were analyzed and visualized using the Omnilog OPM package in R 3.6.3 software [39]. Significant differences in carbon source utilization between isolates were further confirmed by ANOVA test (*P*<0.05).

## Results

### Diversity of M8 isolates recovered from M8 dry powder

To describe the intra-strain diversity in the M8 dry powder, 24 clones of M8 isolated from two sachets of M8 dry powder were selected for deep sequencing using the Illumina HiSeq platform (Supplementary Table 2). On average, 1139.00 Mb of high-quality data were generated for each genome, corresponding to 554.83- to 612.39-fold sequencing depth. Compared with the reference M8 genome retrieved from the NCBI database, all but one isolates carried SNPs. The average number of SNPs in each SNP-possessing isolate was 3.0 SNPs, ranging from one (six isolates) to eight (one isolate) SNPs, suggesting that there was pre-existing intra-strain diversity within the M8 dry powder.

### Homologous M8 isolates recovered from breast milk, maternal, and infant feces

A total of 11 healthy mother-infant pairs during lactation were recruited for this study (Supplementary Table 1). During the trial, the lactating mothers ingested M8 dry powder daily (6×10^10^ cfu/d). Breast milk, feces of mothers and infants were collected continuously once or twice a week for 8–15 weeks after starting M8 intervention (Supplementary Table 1). Over 2800 bacterial colony clones were isolated across all collected samples according to their morphological characteristics. After 16S rDNA identification, a total of 222 isolates of *B. animalis* subsp. *lactis* were recovered from 11 healthy mother-infant pairs, including 28 isolates from breast milk, 148 isolates from maternal feces, and 46 isolates from infant feces. The genomes of these isolates were sequenced on the Illumina HiSeq platform for further phylogenetic and genomic analyses (Supplementary Table 3). On average, 1235.54 Mb of high-quality data were generated for each genome, corresponding to 507.19- to 989.73-fold sequencing depth.

**Fig. 1:**
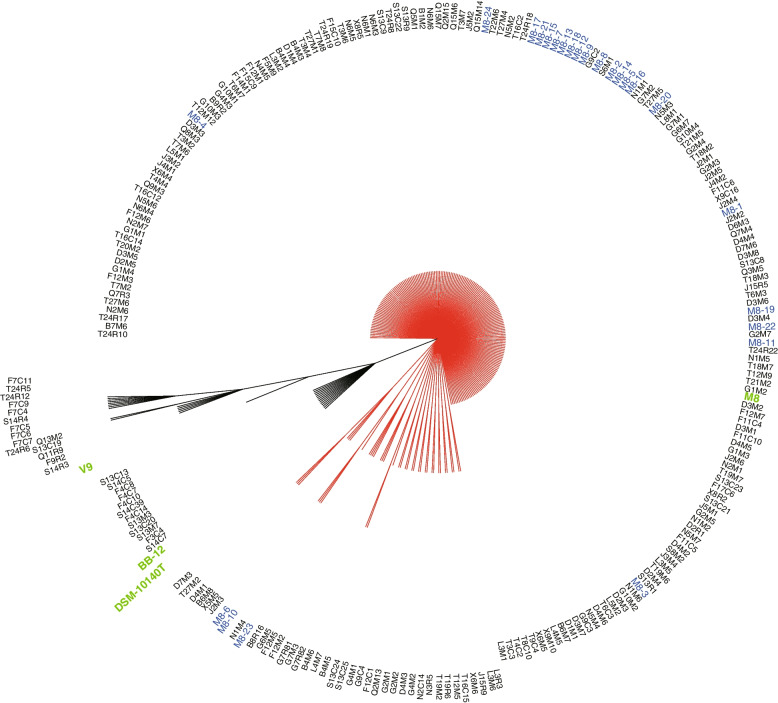
Phylogenetic tree constructed based on the core genes of 250 genomes of *Bifidobacterium animalis* subsp. *lactis*. The tree was built by the maximum likelihood tree method using DNA sequences of 1488 core genes of 250 genomes with 1000 bootstrap iterations. The 250 genomes included 222 isolates of mother-infant pairs (written in black), 24 isolates of M8 dry powder (written in blue), and four reference strains, including M8, BB-12, V9, and DSM 10140T (written in green). The phylogenetic tree was separated into a big (red) and a small (black) branches. Isolates in the big (red) branch were phylogenetically inseparable, which included 195 isolates from mother-infant pairs, all 24 isolates from M8 dry powder, and M8. The small branch comprised 27 isolates from mother-infant pairs, BB-12, V9, and DSM 10140T

To test our hypothesis of the existence of direct transmission of M8 from mother to infant through lactation, a phylogenetic tree was built to reconstruct clonal ancestry based on 1488 core genes of 222 isolates from mother-infant pairs, 24 isolates recovered from M8 dry powder, the original M8 strain, two commercial strains (BB-12 and V9), and the type strain (DSM 10140T) (Fig. 1). Owing to the high inter-strain genome similarity of *B*. *animalis* subsp. *lactis*, differentiating between M8 homologous isolates and other *B*. *animalis* subsp. *lactis* strains intrinsically present in the gut of the participated mothers was anticipated to be a difficult task. Thus, several reference strains were included in this analysis. The phylogenetic tree exhibited imbalanced topology, with a major clade comprising 195 isolates of mother-infant pairs that were phylogenetically inseparable from the M8 dry powder-recovered isolates and M8. The remaining 27 isolates of mother-infant pairs, BB-12, V9, and DSM 10140T did not belong to this clade. Interestingly, the 195 isolates from mother-infant pairs in the major clade were almost identical with M8 at the genomic level, with only very small average (5.2 SNPs) and maximum (11 SNPs) SNP distances. These results indicated that M8 was likely the most recent common ancestor of these 195 isolates from mother-infant pairs and the M8 dry powder isolates, though the chance that these 195 isolates were part of the endogenous gut microbiota of the mothers could not be completely ruled out due to the lack of a negative control group of mothers without taking the probiotic powder.

These 195 M8 homologous clones were isolated from all maternal fecal samples (145 isolates; 11/11), the majority of breast milk samples (21 isolates; 10/11), and around half of the infant fecal samples (29 isolates; 6/11) (Table 1). In other words, homologous isolates of M8 were isolated from the maternal feces of all mother-infant pairs and were notably recovered from all three types of samples of five mother-infant pairs.

**Table 1:** Homologous *Bifidobacterium animalis* subsp. *lactis* Probio-M8 (M8) isolates recovered from mother-infant pairs

Family code	Number of homologous isolates of M8			Total
	Maternal feces	Breast milk	Infant feces	
B	8 (2)^a^	2	─^b^	10
D	22	1	─	23
F	8	─	8	16
G	22 (1)	2	3 (1)	27
J	12 (2)	2	─	14
L	9	1	─	10
N	19	1	1 (2)	21
Q	10 (1)	1	─ (2)	11
S	2	2	7	11
T	28 (2)	7	9	44
X	5	2	1	8
Total	145	21	29	195

^a^The number of metagenome-assembled genomes of homologous M8 from the respective fecal samples is shown in brackets. ^b^ “─” indicates not detected

### Metagenomics confirmed vertical transmission of M8 from mother to infant

**Fig. 2:**
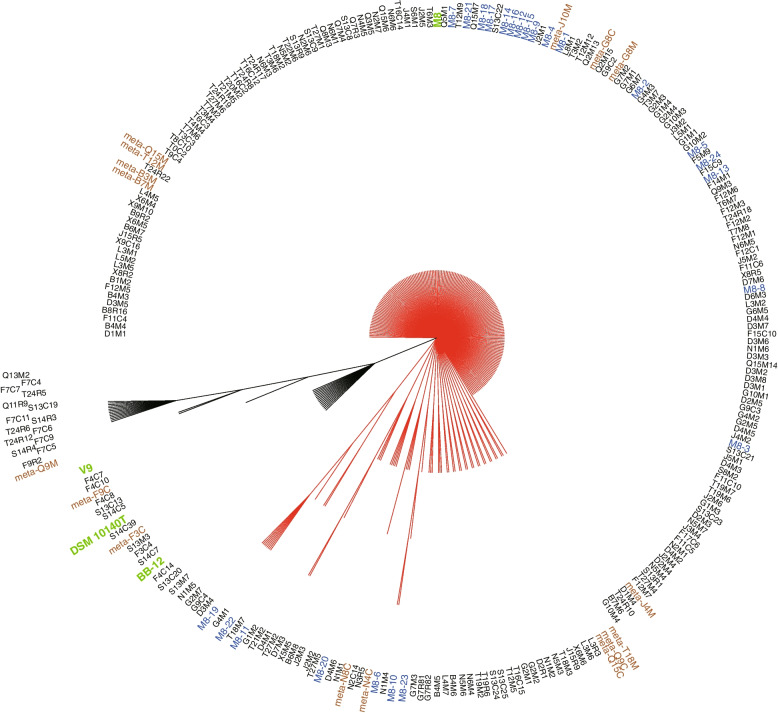
Phylogenetic tree constructed based on the core genes of 266 genomes of *Bifidobacterium animalis* subsp. *lactis*. The tree was built by the maximum likelihood method using DNA sequences of 1271 core genes of 266 genomes with 1000 bootstrap iterations. The 266 genomes included 16 metagenome-assembled genomes (MAGs; prefixed with ‘meta’ and written in brown), 222 isolates of mother-infant pairs (written in black), 24 isolates from M8 dry powder (written in blue), and four reference strains, including M8, BB-12, V9, and DSM 10140T (written in green). The phylogenetic tree was separated into a big (red) and a small (black) branches. Isolates in the big (red) branch were phylogenetically inseparable, including 13 MAGs, 195 isolates from mother-infant pairs, all 24 isolates from M8 dry powder, and M8. The small branch comprised three MAGs, 27 isolates from mother-infant pairs, and genomes of BB-12, V9, and DSM 10140T

Maternal and infant fecal samples collected after 4 and 8 weeks of M8 intake were subjected to deep sequencing (generated ~50 GB sequencing data per sample). A total of 16 high-quality *B. animalis* subsp. *lactis* genomes were assembled by MetaSPAdes (Supplementary Table 4). A second phylogenetic tree was constructed using the same method and parameter settings described above. Apart from the 250 already included genomes, the 16 newly assembled *B. animalis* subsp. *lactis* genomes were also incorporated into the dataset for tree building (Fig. 2). The number of core genes decreased from 1488 to 1271 after adding these MAGs into the dataset.

The topology of the second phylogenetic tree was highly similar to that of the first one (Fig. 1), comprising a large branch and a small branch. Thirteen MAGs were distributed to the large branch and were phylogenetically inseparable from M8 and M8 homologous isolates recovered from M8 dry powder and mother-infant pairs, suggesting that their high homology with M8 strain. Five and eight of the MAGs were assembled from the metagenomes of three infant and five maternal feces samples, respectively. The results obtained by culture-independent method, metagenomics analysis in this case, were largely consistent with those achieved by conventional culture and isolation, though in most mother-infant pairs the laboratory cultivation method recovered a higher number of clones than MAGs. One exception was that M8-related clones were not isolated from the infant feces of family Q, but the metagenomics analysis returned two MAGs.

### Genetic diversity of M8 homologous isolates recovered from mother-infant pairs

**Fig. 3:**
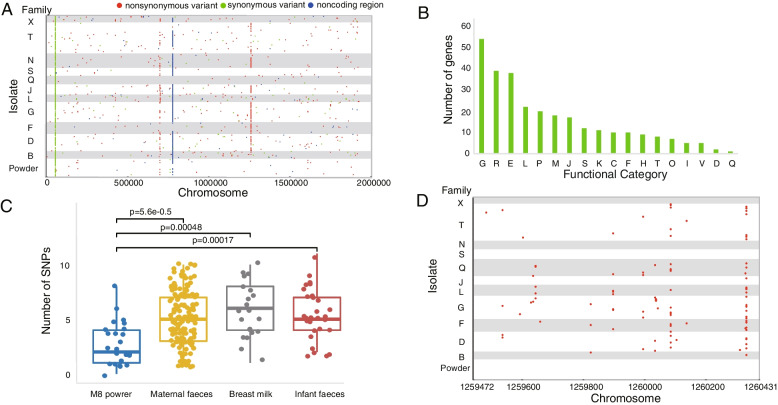
Single-nucleotide polymorphisms (SNPs) identified in M8 homologous isolates of mother-infant pairs. **A** Distribution of SNPs in the chromosome of M8. The white and gray stripes from bottom to top of the plot represent isolates of M8 dry powder (Powder), families B, D, F, G, L, J, Q, S, N, T, and X (family), respectively. **B** Distribution of nonsynonymous SNPs across 18 Clusters of Orthologous Genes (COG) functional categories. The letter below the horizontal ordinate represents the functional category: [G] Carbohydrate transport and metabolism; [R] General function prediction only; [E] Amino acid transport and metabolism; [L] Replication, recombination and repair; [P] Inorganic ion transport and metabolism; [M] Cell wall/membrane/envelope biogenesis; [J] Translation, ribosomal structure and biogenesis; [S] Function unknown; [K] Transcription; [C] Energy production and conversion; [F] Nucleotide transport and metabolism; [H] Coenzyme transport and metabolism; [T] Signal transduction mechanisms; [O] Posttranslational modification, protein turnover, chaperones; [I] Lipid transport and metabolism; [V] Defense mechanisms; [D] Cell cycle control, cell division, chromosome partitioning; [Q] Secondary metabolites biosynthesis, transport and catabolism **C** Number of SNPs detected in isolates from M8 dry powder and samples of mother-infant pairs (maternal feces, breast milk, infant feces). Data were represented as mean ± SEM. *P* values were generated by t-tests. **D** Distribution of SNPs in the *glc*U gene respective to its chromosome location. All SNPs in this gene were nonsynonymous

A total of 499 SNPs were identified in 195 homologous isolates of M8 recovered from mother-infant pairs (Fig. 3A). Among which, nine SNPs were commonly detected among isolates recovered from both the M8 dry powder and mother-infant pairs, including some high-frequency variants, such as SNPs at positions 53,155, 698,170, and 777,230 of the chromosome of M8. The remaining 490 SNPs were exclusively identified in homologous isolates from mother-infant pairs, including 69 in noncoding regions, 100 synonymous variants, and 321 nonsynonymous variants (Supplementary Table 5).

More than 91% of the SNPs (450 SNPs) were identified in only one isolate, indicating that they were random variants. In contrast, the nonsynonymous mutations appeared to be unevenly distributed predominantly to metabolism-related functional genes, especially carbohydrate and amino acid transport and metabolism (Fig. 3B). Significantly more SNPs were detected in isolates from mother-infant pairs than those from the M8 dry powder (*P*<0.01), and no significant difference was found among maternal feces, infant feces, and breast milk, implicating that mutations mainly occurred in the mothers (Fig. 3C).

Interestingly, a relatively high proportion (20/321; 6.23%) of nonsynonymous variations was notably concentrated in the *glc*U gene, a 960 bp gene that encoded a sugar transporter (M8PIadj_1109, position on the chromosome of M8 strain: 1259472 to 1260431). The detected nonsynonymous variants were verified by PCR (data not shown). Intriguingly, these variants were found exclusively in isolates of the mother-infant pairs (96 of 195 isolates from eight mother-infant pairs; 77, 15, and four isolates from maternal feces, infant feces, and breast milk, respectively), but not any of the 24 M8 dry powder-associated isolates (Fig. 3D; Supplementary Table 6). Although the gene exhibited 20 nonsynonymous mutations at various locations, each isolate was found to contain mutation only at one site. The mutations occurred preferentially at some specific positions. For example, 20 and 29 mutations were detected at the genome positions of 1,260,085 and 1,260,332, respectively, which were significantly more frequent than any other loci (Fig. 3D).

In addition to *glc*U, some high-frequency mutation sites were also found in the mother-infant isolates (Supplementary Table 5). More than five isolates exhibited point mutation at each of the top six high-frequency mutation sites, including four nonsynonymous mutations, one synonymous mutation, and one intergenic region mutation. The nonsynonymous mutation at the genome position of 700,045 was identified in 18 isolates from five mother-infant pairs (11, one, and six isolates from maternal feces, infant feces, and breast milk, respectively). The mutation was located on a phosphatidylglycerol lysyltransferase-encoding gene, and its gene product is responsible for the biosynthesis of lysyl-phosphatidylglycerol [40]. The other three nonsynonymous mutations (at the genome positions of 203,327, 424,251, and 1,916,533) were identified in six, eight, and six isolates, respectively. The three mutations were located on genes encoding a branched-chain amino acid ABC transporter substrate-binding protein, a 3-deoxy-7-phosphoheptulonate synthase, and a lytic transglycosylase, respectively. Whether these mutations were related to environmental adaptation needs further study.

### Enhanced ability of carbon source utilization in mutants

**Fig. 4:**
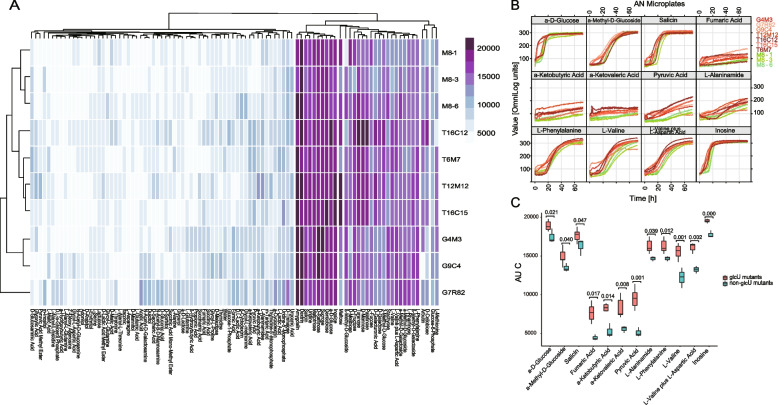
Comparison of efficiency of carbon source utilization of mutants harboring *glc*U gene variations with those without in Phenotype MicroArray analysis on Biolog AN microplates. **A** Cluster heatmap of area under curve (AUC) of respiration kinetics curves of each substrate of 10 isolates (seven mother-infant pair isolates containing nonsynonymous mutation in the *glc*U gene: G4M3, G9C4, G7R82, T6M7, T12M12, T16C12, T16C15; and three M8 dry powder-associated isolates without mutation in the *glc*U gene: M8-1, M8-3, M8-6). Cluster analysis was performed by the unweighted pair group method with arithmatic mean (UPGMA). The color scale represents AUC. A higher value represents a larger AUC. **B** The respiration kinetics curves of 12 differential metabolic carbon sources of the 10 isolates. Time in hours and the observed metabolic signals in OmniLog units are represented on the *x*- and *y*-axes, respectively. **C** Metabolic differences in the capacity of utilization of 12 carbon sources between mother-infant pair isolates containing mutation in *glc*U (*glc*U mutants) and M8 dry powder-associated isolates without mutation in *glc*U (non-*glc*U mutants). Data were represented as mean ± SEM. *P* values were generated by *t* test

Since a significant proportion of nonsynonymous mutations occurred at genes related to metabolism-related genes, especially *glc*U, changes in the mutants’ ability of carbon utilization were investigated. Specifically, the efficiency of carbon utilization of 95 conventional sugars and organic acids of seven *glc*U-mutant isolates (G4M3, G9C4, G7R82, T6M7, T12M12, T16C12, and T16C15 from two mother-infant pairs) was compared with several isolates of M8 dry powder (M8-1, M8-3, and M8-6) not exhibiting mutation in *glc*U using Biolog Phenotype MicroArray for a period of 72 h (Fig. 4A; Supplementary Fig. 1). The efficiencies of utilization of most carbon sources were highly similar across the three M8 dry powder-associated isolates, reflected by their highly alike patterns of respiratory kinetics curves when different carbon sources were tested. In contrast, comparing with the non-mutated isolates, the mutant isolates showed obvious variations in their growth responses towards different carbon sources. An interesting clustering pattern was observed in the carbon utilization profile (Fig. 4A). The clustering pattern implicated a higher similarity in the carbon utilization profile among the three mutant isolates of family G than those of family T and the M8 dry powder-associated isolates. The four mutant isolates of family T and the M8 dry powder-associated isolates formed two subclusters, respectively, suggesting obvious differences in the carbon utilization capacity between these two groups of isolates. Our results reflected that the isolates from the same mother-infant pairs had more similar carbon utilization preference compared with isolates from non-related individuals.

The kinetic bacterial growth curves of 12 specific carbon sources, including the basal substrates (a-d-glucose and a-methyl-d-glucoside) and organic acids (fumaric acid, a-ketobutyric acid, a-ketovaleric acid, pyruvic acid, L-alaninamide, L-phenylalanine, L-valine, L-valine plus L-aspartic acid, salicin, and inosine), showed apparently better growth of mutant isolates compared with M8 dry powder-associated isolates (Fig. 4B), and evidenced by a shorter time required for reaching the stationary phase (e.g., a-D-glucose) or a larger AUC during/at the end of the monitoring period (e.g., L-alaninamide, a-ketobutyric acid, a-ketovaleric acid, and pyruvic acid). Notably, it was hard to identify a classical bacterial growth pattern in the growth curves of some substrates (e.g., fumaric acid, a-ketobutyric acid, a-ketovaleric acid, pyruvic acid, and L-alaninamide) because of the lack of a distinctive lag phase and failure in reaching the stationary phase, suggesting suboptimal growth. However, the observation that most mutant isolates exhibited stronger signals when grown in these substrates implicated their enhanced capacity in catabolizing and utilizing substrates that were normally less preferentially used by isolates without mutation in the *glc*U gene. The overall AUCs of the mutant isolates were larger than those of the M8 dry powder-associated isolates when grown in these 12 carbon sources (*P*<0.05, ANOVA; Fig. 4C), indicating that these substrates enhanced the growth of the mutant isolates relative to the non-mutants. These results together suggested that the nonsynonymous mutations in *glc*U indeed improved the carbon metabolism capacity and efficiencies in utilizing normally less preferred organic acid substrates.

## Discussion

A large body of literature supports that mothers’ microbiota could be transmitted vertically to infants; however, direct evidence, particularly on a fine taxonomic level, is still lacking. Thus, this study tested the hypothesis of translocation of maternally ingested bacteria to infant gut via lactation. Eleven healthy mother-infant pairs during lactation were recruited, and the lactating mothers ingested M8 dry powder daily. Samples of maternal feces, breast milk, and infant feces were collected every week for recovery of the biomarker M8 strain using both traditional culture methods and high-throughput sequencing. This work provided direct and strain-level evidence via tracking the biomarker M8 strain in breast milk and fecal samples of mother-infant pairs after maternal intake. Intra-strain diversity and metabolic phenotype analyses further showed that these bacteria could adapt to new environmental niches via genomic polymorphism, especially in the sugar transporter *glc*U gene.

This study combined both traditional culture method and high-throughput sequencing to confirm vertical transmission of M8 after maternal intake. One pre-requisite for successful detection of M8 homologous isolates for both methods was that adequate amounts of target bacteria must be present in the samples. Since the presence of large quantities of target bacteria in the samples was not anticipated, strain recovery and identification were challenging and had become probabilistic events [41]. To maximize the chance of recovering M8 homologous bacteria, the amount of M8 ingested daily was rather high (6×10^10^ cfu/d), which was several or even tens of times higher than the recommended daily oral dose of other similar probiotic products available on the market. The application of a high probiotic dose was crucial in enhancing the detection of the target strain. Indeed, in our preliminary study, a standard daily dose (1×10^10^ cfu/d) was used, which returned a non-statistically significant number of recovered M8 homologous strains (data not shown), even though vertical transmission of M8 could still have occurred. Apart from increasing the probiotic dose, extensive effort was also devoted to picking an enormous number of clones for further testing. Indeed, over 2800 bacterial colony clones were manually picked and cultured for further taxonomic identification, and only 222 among which were confirmed to be *B. animalis* subsp. *lactis*. Despite the serious and multiple attempts made to recover M8 homologous clones from all samples of the 11 mother-infant pairs, five infant fecal samples and one breast milk did not return any target bacterial clones. The results of successful recovery of M8 clones from all 11 maternal fecal samples and the majority of breast milk samples, however, suggested that our procedures were effective and technically correct. Similarly, even though a large amount of metagenomic data (~50 G per sample, i.e., about ten times of data amount compared with routine metagenomic studies) was obtained using deep sequencing, M8 homologous MAGs were successfully assembled from samples of only a low number of families (maternal and infant fecal samples of five and three families, respectively). These results consistently suggested that only a minuscule amount of M8 homologous bacteria was present in the samples, and they could be under the detection limits of the applied methods in some cases.

This work was performed at a time frame partly overlapping with the start of the COVID-19 pandemic, making it even harder to recruit eligible mother-infant pairs. Finally, only 11 healthy mother-infant pairs during lactation were recruited and completed the study. Owing to the low number of recruited participants, a negative control group of women not taking the probiotic was not incorporated into the study design. Thus, a baseline compositional profile of *B*. *animalis* subsp. *lactis* in the fecal and breast milk samples of subjects of no probiotic intake was not available for comparison with data obtained from subjects consumed the probiotic powder, weakening the conclusions drawn in this study. Nevertheless, interesting trends were observed. It was not surprising that all maternal fecal samples were positive for M8 homologous isolates, as all participated mothers consumed a large of amount of active M8 daily during the course of this work. Especially, M8 was previously shown to have a strong tolerance to and a high survival rate in the transit through the human gastrointestinal tract [21]. The high frequency of M8 homologous isolates recovered from most breast milk samples (10/11; 90.9%) strongly supported that oral- and entero-mammary routes of bacterial translocation exist and are common to most individuals, and the existence of similar mechanisms of bacterial translocation has previously proposed [18, 42]. There is no doubt that milk microbiota is present; however, the impact of milk microbiota on infant gut colonization is still an unresolved question [43], which could be partially answered by simultaneously tracking M8 homologous bacteria in the infant fecal sample counterparts. Three different presence-absence combinations were observed. Firstly, most (6/11; 54.5%) of the mother-infant pairs contained M8 homologous isolates (cultured clones and/or MAGs) in all three types of samples. In this case, it is logical to postulate that M8 homologous bacteria gained access to the infant gut via intake of the breast milk. Secondly, four mother-infant pairs (4/11; 36.4%) contained M8 homologous isolates in the samples of maternal feces and breast milk but not the infant feces counterparts. The absence of positive clones in the infant feces could simply be a result of the scantiness of target bacteria to be detected or was really due to biological reasons leading to the inability of survival and/or colonization of M8 homologous bacteria in the gut of these individuals. The success in bacterial colonization has been reported to be a personalized process linking to both host factors and microbiome features [44]. The final combination occurred only in family F, in which positive clones were detected in both maternal and infant feces but not in breast milk samples. Such results raised the question of whether M8 homologous bacteria gained access to the breast milk at all. The fact that only a small number of positive clones were detected in the breast milk samples of almost all mother-infant pairs suggested that M8 homologous bacteria were present only in a very low abundance. Thus, it was not impossible that the failure in detecting the biomarker bacteria in the breast milk samples of family F was simply due to the low probability of picking the right clones from the scant amount of target bacteria present, which could be far below the detection limit. In addition, the “screening” strategies employed in this study for bacterial detection was laborious. Unfortunately, the biomarker strain of this study was *B. animalis* subsp. *lactis*, and strains in this subspecies are highly conserved [45]. The very high inter-strain genome similarity made it very difficult to differentiate between M8 homologous isolates and alike strains intrinsically present in the gut of the participated mothers. Furthermore, the applied M8 strain did not carry any distinctive markers to aid differentiation from other highly alike strains, and provided the nature of this trial, it was not possible to introduce an exogenous genetic biomarker for such purpose. On the other hand, it was also possible that the vertical transmission in this case was via alternative and yet to be identified path other than the proposed oral- and entero-mammary routes.

Another interesting observation is the high number of isolated M8 homologous clones that adopted genomic adaptations in response to the change to a human gut environment, revealed by analyses of their diversity and carbon utilization preference and capacity. Although the 24 M8 dry powder-associated isolates did exhibit SNPs and intra-strain diversity, their number of SNPs and diversity were significantly lower than the mother-infant isolates. The SNPs adopted by the mother-infant isolates occurred mostly in genes related to carbohydrate and amino acid transport and metabolism, which is strongly suggestive of genomics-based metabolic adaptation in nutrient/carbon acquisition and utilization for better survival. Such speculation is supported by the observation of high frequencies of non-synonymous mutations in the *glc*U gene that encoded a sugar transporter responsible for glucose uptake. The mutation frequency of this gene was as high as 49.2% across 195 mother-infant isolates. It was detected in eight mother-infant pairs, implicating extremely strong directional selection pressure. Consistently, phenotypic analysis of carbon utilization of these mutants revealed significant enhancement in their metabolic/growth rate compared with those without SNPs in *glc*U gene. In addition, the ability of the mutant isolates to utilize 12 common carbon sources, such as fumaric acid, inosine, and valine, improved significantly, showing an extensive metabolic repertoire. Such metabolic shift is more in line with the characteristics of the human gut microbiota [46]. In most laboratory cultivation or industrial production process, sufficient glucose is provided as the major carbon source to optimize bacterial growth. In contrast, in the human digestive system, the majority of simple sugars including glucose are absorbed in the small intestine [47, 48], and the lower gut is deprived of glucose and other simple sugars. Thus, to enhance the competitiveness and survival, it is likely that, after gaining access to the mother’s gut, the M8 strain was environmentally driven to expand its metabolic repertoire, so as to increase its efficiency in utilizing intermediate products generated in fermentation by other bacteria as carbon source. Especially, it is known that metabolic cross-feeding often occurs between bifidobacteria and the gut microbiota [49].

One potential concern of the current study is contamination of samples of mother-infant pairs during sample handling and collection by the participating mothers. As subjects took M8 dry powder every day, there might be elevated risk of contamination of the skin or surrounding air by M8. Indeed, such risk was carefully considered prior to the initiation of this work. Thus, participants were educated of the proper way of sample collection and handling before this trial started, and the sampling operation was standardized to avoid contamination as much as possible. However, the chance of contamination could not be completely ruled out [50]. On the other hand, the different SNV patterns and the high frequency of mutations in the *glc*U gene of the mother-infant isolates implicated their divergence from the M8 dry powder-associated isolates, counterarguing the viewpoint of sample contamination.

The strength of this study is the application of state-of-the-art technologies, ranging from conventional microbiology to metagenomics, to address an interesting topic, i.e., the initial colonization of the infant gut by bacteria transmitted by the mother, from a unique perspective of genomics and metabolic adaptations of the ingested strain in host colonic environment. Our hypothesis was validated in detail, exploiting also golden-standard in silico and in vitro approaches. The findings of this study deepen the understanding of mother-infant bacterial transmission and have a direct impact on infant nutrition, especially on how milk microbiota or maternal probiotic intake during lactation shapes infant gut colonization, providing insights into both basic science and industrial probiotic product development.

Bifidobacteria are some of the earliest human gut colonizers, which possess specific carbohydrate metabolism enzymes that are otherwise not present in the human body. These enzymes help decompose human milk oligosaccharides in breast milk, facilitating nutrient digestion and absorption [51]. A previous study showed that the lower the proportion of bifidobacteria colonized in early infants, the higher the probability of an unbalanced gut microbiota then and in later life [8]. In addition, bifidobacteria in newborns could help maintain immune homeostasis and inhibit excessive immune response [52]. Therefore, appropriately supplementing bifidobacteria to newborns is beneficial to both short-term and long-term health, and compared with direct probiotic supplementation, the intake of bifidobacteria by mothers via breastfeeding is undoubtedly safer and more effective.

## Conclusions

By using M8 as a biomarker strain, this study demonstrated that small amounts of maternally ingested bacteria could translocate to the infant gut via oral- and entero-mammary routes in more than half of the mother-infant pairs, suggesting it is feasible to nourish infants with bifidobacteria and possibly other probiotic bacteria via lactation. A number of exciting questions regarding the vertical transmission pathway and its potential applications remain to be answered, e.g., whether these were the only routes of mother-to-infant bacterial transmission and the exact trajectories of bacterial translocation, why individual difference was observed in the outcome of bacterial colonization, if there was species/strain preference/specificity in the access to this pathway and finally colonization of the infant gut, if any correlation existed between the amounts of ingested and detected probiotic cells, and how the efficiency of the process could be enhanced. Nonetheless, our findings provided insights into the impact of milk microbiota on infant gut colonization and alternative means of supplementing probiotics to newborns.

## Supplementary Information

**Additional file 1: Supplementary Table 1.** Information of 11 healthy mother-infant pairs and sample collection. **Supplementary Table 2.** Genome information of 24 isolates recovered from M8 dry powder. **Supplementary Table 3.** Genome information of *Bifidobacterium animalis* subsp. *lactis* recovered from mother-infant pairs. **Supplementary Table 4.** Information of metagenome-assembled *Bifidobacterium animalis* subsp. *lactis* genomes. **Supplementary Table 5.** Information of single nucleotide polymorphisms (SNPs) identified in 195 homologous isolates of M8 recovered from mother-infant pairs. **Supplementary Table 6.** Information of single nucleotide polymorphisms (SNPs) in the *glc*U gene. **Supplementary Table 7.** Information of metagenomic data of maternal and infant fecal samples.

**Additional file 2: Supplementary Figure 1.** The respiration kinetics curves of Phenotype MicroArray analysis for 10 isolates (seven mutant isolates and three M8 dry powder-associated isolates) on Biolog AN microplates. Time in hours and the observed metabolic signals in OmniLog units are represented on the x- and y-axes, respectively.

## Data Availability

The whole-genome sequences of the four *B. animalis* subsp. *lactis* reference strains, namely M8, V9, BB-12, and DSM 10140T, were retrieved from DDBJ/ENA/GenBank database under the accession numbers CP047190, CP001892, CP001853, and CP001606, respectively. The genomic and metagenomic data generated by this work were deposited in \DDBJ/ENA/GenBank database under the Bioproject accessions PRJNA819840 (Supplementary Table 2), PRJNA819217 (Supplementary Table 3), and PRJNA810575 (Supplementary Table 7), respectively.

## Abbreviations

AUC: Area under the curve
COG: Clusters of orthologous genes
InDels: Insertions and deletions
KEGG: Kyoto Encyclopedia of Genes and Genomes
M8: *Bifidobacterium animalis* subsp. *lactis* Probio-M8
MAGs: Metagenome-assembled genomes
PBS: Phosphate buffer saline
RCM: Reinforced clostridial medium
SNPs: Single-nucleotide polymorphisms
